# Biophysical analysis of HTLV-1 particles reveals novel insights into particle morphology and Gag stoichiometry

**DOI:** 10.1186/1742-4690-7-75

**Published:** 2010-09-20

**Authors:** Iwen F Grigsby, Wei Zhang, Jolene L Johnson, Keir H Fogarty, Yan Chen, Jonathan M Rawson, Aaron J Crosby, Joachim D Mueller, Louis M Mansky

**Affiliations:** 1 Institute for Molecular Virology, University of Minnesota, Minneapolis, MN 55455, USA; 2 Department of Diagnostic and Biological Sciences, School of Dentistry, University of Minnesota, Minneapolis, MN 55455, USA; 3 Department of Microbiology, Medical School, University of Minnesota, Minneapolis, MN 55455, USA; 4 School of Physics and Astronomy, University of Minnesota, Minneapolis, MN 55455, USA

## Abstract

**Background:**

Human T-lymphotropic virus type 1 (HTLV-1) is an important human retrovirus that is a cause of adult T-cell leukemia/lymphoma. While an important human pathogen, the details regarding virus replication cycle, including the nature of HTLV-1 particles, remain largely unknown due to the difficulties in propagating the virus in tissue culture. In this study, we created a codon-optimized HTLV-1 Gag fused to an *EYFP *reporter as a model system to quantitatively analyze HTLV-1 particles released from producer cells.

**Results:**

The codon-optimized Gag led to a dramatic and highly robust level of Gag expression as well as virus-like particle (VLP) production. The robust level of particle production overcomes previous technical difficulties with authentic particles and allowed for detailed analysis of particle architecture using two novel methodologies. We quantitatively measured the diameter and morphology of HTLV-1 VLPs in their native, hydrated state using cryo-transmission electron microscopy (cryo-TEM). Furthermore, we were able to determine HTLV-1 Gag stoichiometry as well as particle size with the novel biophysical technique of fluorescence fluctuation spectroscopy (FFS). The average HTLV-1 particle diameter determined by cryo-TEM and FFS was 71 ± 20 nm and 75 ± 4 nm, respectively. These values are significantly smaller than previous estimates made of HTLV-1 particles by negative staining TEM. Furthermore, cryo-TEM reveals that the majority of HTLV-1 VLPs lacks an ordered structure of the Gag lattice, suggesting that the HTLV-1 Gag shell is very likely to be organized differently compared to that observed with HIV-1 Gag in immature particles. This conclusion is supported by our observation that the average copy number of HTLV-1 Gag per particle is estimated to be 510 based on FFS, which is significantly lower than that found for HIV-1 immature virions.

**Conclusions:**

In summary, our studies represent the first quantitative biophysical analysis of HTLV-1-like particles and reveal novel insights into particle morphology and Gag stochiometry.

## Introduction

There are approximately 15-20 million people infected by human T-lymphotropic virus type 1 (HTLV-1) worldwide [1]. HTLV-1 infection can result in a number of severe disorders including adult T cell leukemia/lymphoma (ATLL) as well as HTLV-1 associated myelopathy/tropical paraparesis (HAM/TSP) [2,3]. Despite its association with cancer and its significant impact on human health, many of the details regarding the replication, assembly and fundamental virus particle structure remain poorly understood.

The Gag polyprotein is the main retroviral structural protein and is sufficient, in the absence of other viral proteins, for the production and release of immature VLPs [4]. The Gag polyprotein is composed of three functional domains: matrix (MA), caspid (CA), and nucleocapsid (NC). Typically, upon budding or immediately after immature particle release, proteolytic cleavage of the Gag polyproteins takes place and results in virus particle core maturation. The Gag polyprotein is cleaved into MA, CA, and NC by the viral protease. The newly processed proteins reorganize into structurally distinct mature virions: MA remains associated with the viral membrane; CA undergoes conformational changes and reassembles into a viral core, which encapsulates a complex of NC, genomic RNA, and other important viral proteins [5-7].

Studies with many retroviruses, including human immunodeficiency virus type 1 (HIV-1), have shown that retroviral assembly is initiated by binding the myristoyl moiety of MA with lipid rafts at the plasma membrane [8-11]. The MA-membrane interaction is thought to stimulate Gag oligomerization, the interaction between viral genomic RNA and NC, and the recruitment of a variety of host factors. Accumulation of Gag at the plasma membrane triggers the activation of the ESCRT machinery which creates the membrane curvature that results in the budding of immature virus particles [12]. Analysis of Gag molecules in immature HIV-1 particles have revealed that the MA domain is located at the membrane with the CA and NC domains projecting towards the center of the particle [13].

Cryo-electron tomography (cryo-ET) combined with three-dimensional (3D) reconstructions have provided highly detailed structural information for HIV-1. Structural studies have revealed that HIV-1 Gag proteins form an incomplete paracrystalline lattice in immature particles [14,15]. This incomplete Gag lattice was observed to consist of a hexameric organization with 80-Å distance between neighboring ring-like structures [14,15]. While the myristoyl moiety of MA appeared to be associated with membrane, the hexameric ring structure in the 3 D maps were attributed to CA, and the Gag-Gag interactions in the immature particles were proposed to be primarily stabilized by CA and SP1, rather than the affinity of membrane-binding via MA [15].

Despite limited amino acid sequence homology among different retroviruses, the atomic tertiary structures of individual Gag domains exhibit high similarity [16-18]. Therefore, structural and assembly mechanisms of HIV-1 are generally used as a reference model for other retroviruses. However, structural evidence indicates that the conservation of Gag organization between HTLV-1 and HIV-1 is poorly understood. In this study, we have performed cryo-TEM on HTLV-1-like particles. Our study is the first to study HTLV-1 particles in their native, hydrated state. Our results demonstrate an average HTLV-1 particle diameter of ~ 73 nm, which is smaller than previously predicted based on conventional negative staining TEM [19]. Using the novel biophysical technology of FFS, we further demonstrate that there are ~ 510 copies of Gag per HTLV-1 particle, a number that is significantly lower than what is typically found in HIV-1 particles. Finally, our cryo-TEM images analysis reveals a less ordered Gag structure compared to that reported for HIV-1, suggesting that the HTLV-1 Gag shell has a distinct architecture.

## Results

### Creation of a tractable and robust system for the production of HTLV-1-like particles

Previous molecular analyses of HTLV-1 replication have been severely hampered by the fragility of HTLV-1 proviral sequences as well as the low levels of viral replication in tissue culture. Given the technical and experimental limitations of working with HTLV-1, we first sought to create an experimental model system that would be amenable to successfully and efficiently analyze HTLV-1 Gag trafficking and virus particle assembly and release. It is well-established that retroviral Gag polyprotein is sufficient for the assembly and release of VLPs [reviewed by [20]]. Our previous studies indicated that HTLV-1 Gag constructs express Gag at low levels (Huating Wang and Louis Mansky, unpublished observations), presumably due to missing *cis*-elements on the RNA transcript required for efficient nuclear export.

**Figure 1:**
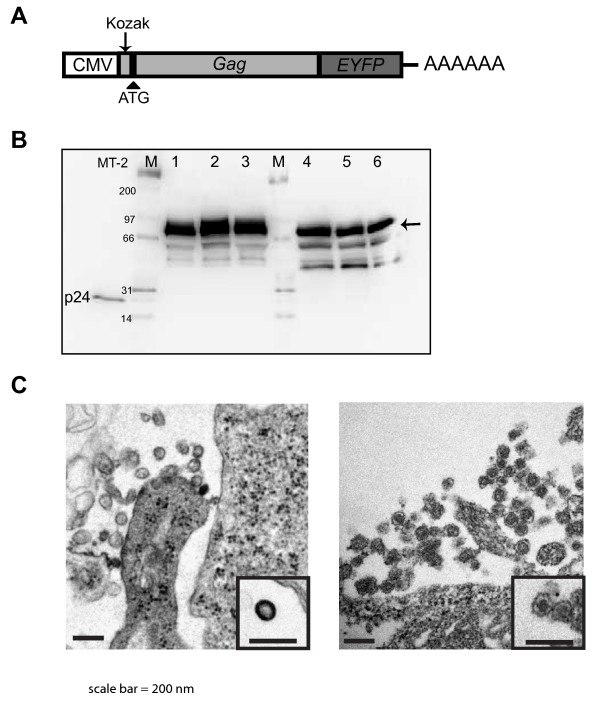
**Development of a model system for the efficient expression of HTLV-1 Gag and robust production of VLPs**. (A). HTLV-1 Gag expression construct. The HTLV-1 Gag gene was codon-optimized with the insertion of a Kozak consensus sequence (arrow) upstream of the ATG start codon (arrowhead). The *EYFP *gene was inserted in-frame prior to the Gag gene stop codon. The CMV promoter and 3'-end poly A are indicated. (B). Immunoblot analysis of HTLV-1 Gag. An anti-HTLV-1 p24 monoclonal was used to detect HTLV-1 Gag-EYFP (arrow). Cell culture supernatants were collected from MT-2 cells was used as a positive control. Lane 1-3 are cell culture supernatants from three independent experiments in which pEYFP-N3-HTLV-1 Gag was transiently transfected into 293T cells; lane 4-6 are the cellular lysates. Lane "M", molecular markers. (C). Transmission electron microscopy of VLPs. Left panel, VLPs produced from 293T cells transiently transfected with pEYFP-N3-HTLV-1 Gag; right panel are HTLV-1 particles from MT-2 cells. Scale bar = 200 nm.

In order to create a tractable and robust system for Gag expression and virus-like particle production, we designed and created a codon-optimized HTLV-1 Gag construct to improve HTLV-1 Gag expression. In order to readily detect Gag expression, trafficking, and incorporation into VLPs, we fused the EYFP to the C-terminal end of the Gag protein. Figure 1A shows the HTLV-1 Gag-EYFP expression construct. In this construct, the Gag-*EYFP *is expressed from a CMV promoter, and a Kozak consensus sequence was engineered upstream of the start codon to facilitate translation initiation as well as an in-frame insertion of the *EYFP *gene sequence just prior to the HTLV-1 Gag gene stop codon. The plasmid is quite stable and readily amplified in *E. coli *(data not shown).

To confirm expression of the fusion construct, 293T cells were transiently transfected with three independent clones of pEYFP-N3 HTLV-1 Gag in parallel experiments. Thirty-six hours post-transfection, HTLV-1 Gag-EYFP protein expression was examined from both cell culture supernatants (Figure 1B, lane 1-3) and from cellular lysates (Figure 1B, lane 4-6). The Gag precursor-EYFP fusion protein, with a molecular mass of approximately 80 kDa was very readily observed, with each of the 3 clones analyzed expressing very high and comparable levels of HTLV-1 Gag-EYFP. The minor bands of smaller molecular mass likely represent partially degraded HTLV-1 Gag-EYFP and not cleavage products of the viral protease, since it is not present in the Gag expression construct. The Gag-EYFP observed in VLPs was primarily full length (Figure 1B, lane 1-3), with undetectable levels of mature capsid (p24) protein.

To investigate the morphology of the particles produced from cells expressing pEYFP-N3 HTLV-1 Gag, transiently transfected 293T cells were harvested and examined by TEM. MT-2 cells, a T-cell line chronically infected by HTLV-1, were examined as a control. As shown in Figure 1C, VLPs can be observed from 293T cells transiently transfected with the pEYFP-N3 HTLV-1 Gag construct (Figure 1C, left panel). In comparison to HTLV-1 produced from MT-2 cells (Figure 1C, right panel), the VLPs produced from the fusion construct resemble immature particles. In particular, the intense electron density along the lipid bilayer of VLPs likely represents the accumulation of Gag-EYFP (Figure 1C, left inset) in contrast to the mature viral cores observed with HTLV-1 particles from MT-2 cells (Figure 1C, right inset).

**Figure 2:**
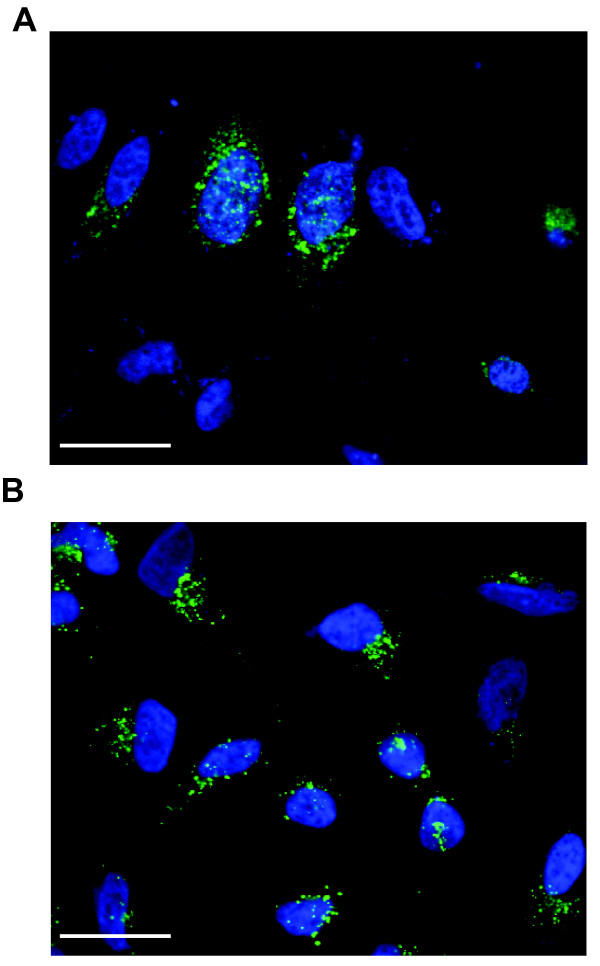
**Cellular localization of HTLV-1 Gag-EYFP and HTLV-1 Gag**. HeLa cells were transiently transfected with pEYFP-N3-HTLV-1 Gag (A) or a HTLV-1 molecular clone (B). The locations of nuclei were identified by DAPI staining (blue), HTLV-1 Gag (green). Scale bars = 28 μm.

We also examined the cellular localization of the Gag-EYFP compared to Gag produced from a HTLV-1 molecular clone. The pEYFP-N3 HTLV-1 Gag construct was transiently transfected into HeLa cells, and 36 hours post transfection, cells were fixed and analyzed by confocal microscopy (Figure 2A, B). Comparable punctate localization of Gag was observed for both the Gag-EYFP and the Gag expressing from the full-length molecular clone. Our observations suggest that Gag-EYFP expression in cells results in an intracellular localization pattern like that of Gag produced from a HTLV-1 molecular clone. In total, our findings provide evidence this construct results in the robust expression of HTLV-1 Gag as well as the highly efficient production of HTLV-1-like particles.

### Analysis of HTLV-1-like particle morphology by cryo-TEM

**Figure 3:**
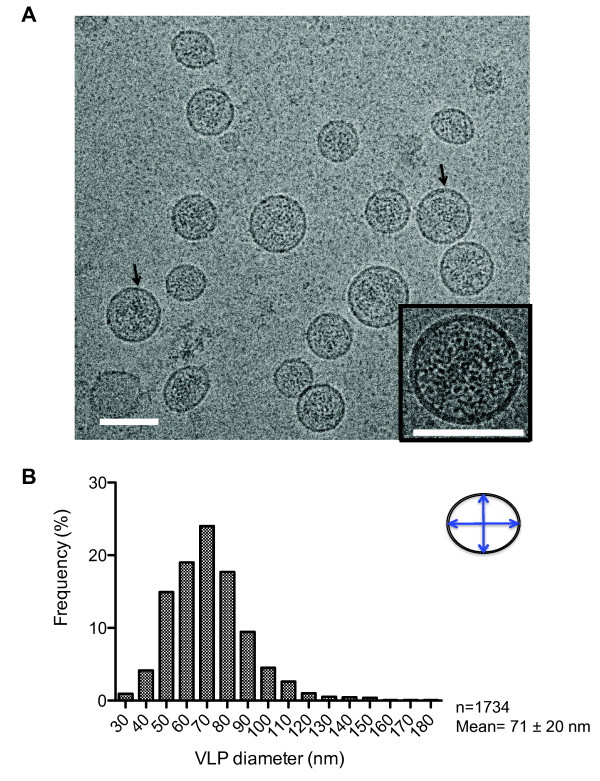
**Cryo-TEM analysis of HTLV-1 Gag-based VLPs**. (A). Cryo-TEM images of VLPs produced from 293T cells. Examples of VLPs that have partially occupied inner electron density are indicated with arrows. The inset shows a magnified view of a representative VLP. Scale bars = 100 nm. (B). Distribution of VLP diameter. Particle diameter was determined by averaging the longest and shortest measurements as indicated in the diagram at the top right corner using the ImageJ software. A total of 1734 VLPs were examined (mean = 71 +/- 20 nm).

To further characterize the VLPs produced from the HTLV-1 Gag-EYFP expression construct, we examined the VLP morphology by cryo-TEM. Supernatants from 293T cells transiently-cotransfected with the HTLV-1 Gag-EYFP expression construct and a VSV-G construct were harvested, concentrated, and then subjected to a 10-40% linear sucrose gradient. The resulting VLPs were then used in cryo-TEM. As shown in Figure 3A, the majority of the resulting VLPs were found to be spherical, with less than 20% of the population showing an elongated morphology. Another example of the particles we observed in our study is shown in Additional file 1. Interestingly, VLPs produced in the absence of an envelope protein resulted in VLPs with irregular shapes, suggesting that the envelope protein helped to stabilize the VLP membrane (data not shown). We used the cryo-TEM images to next measure the diameter of the VLPs, where the average diameter was based on two measurements (as illustrated in Figure 3B), with a total of 1734 particles examined. Similar to other retroviruses, there was a range of particle size. For completeness, we counted all particles that were spherical in shape that appeared to have an electron dense interior. Using these criteria, a total of 1734 particles were examined, ranging from 30 to 237 nm. While the overall range of particles observed was quite wide, the smallest (i.e., under 40 nm) and largest (i.e., over 170 nm) particles represented less than 1% of the total number of particles observed, and their inclusion had little impact on the mean particle size (i.e., 71 +/- 20 nm versus 72 nm +/- 18). We observed that over 25% of the total population was in the 70-80 nm range, with a mean particle size of 71 +/- 20 nm.

### Analysis of VLP radial profile

We next used the information obtained by cryo-TEM to examine the VLP radial profile. For the majority of VLPs, cryo-TEM revealed that the inner Gag structure was indistinguishable (Figure 3A). The partially ordered Gag lattice can be observed (data not shown), although the structure is less obvious compared to that reported for HIV-1 immature particles [13]. Furthermore, the inner density appears to vary among VLPs, with some exhibiting homogenous inner density, while others seem to have an uneven distribution of electron densities attributable to Gag (Figure 3A arrow).

**Figure 4:**
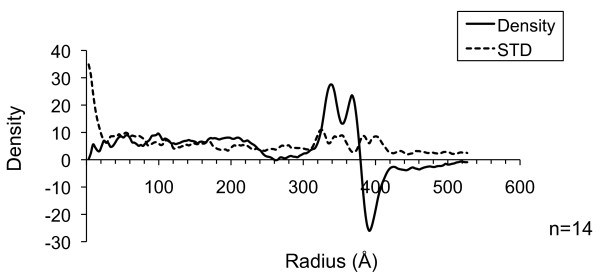
**Radial density profile of the HTVL-1-like particles**. The solid line represents the average density measured; dashed line indicates the standard deviation (n = 14).

To further analyze the electron density of VLPs, we investigated the radial density profile of VLPs. First, the average radial density profile was determined for several particles whose diameters ranged between 70-80 nm. As shown in Figure 4, the average distance between the highest density peaks of inner and outer leaflets of viral membrane with MA domain is approximately 30-Å. The MA domain is indistinguishable from the inner layer of membrane. The electron density profile approaching the center of the particle is relatively flat, suggesting a homogenized inner density. Our observations indicate that the HTLV-1-like particles are quite distinct from those produced from HIV-1 Gag.

### FFS measurement of VLP size and Gag copy number

**Figure 5:**
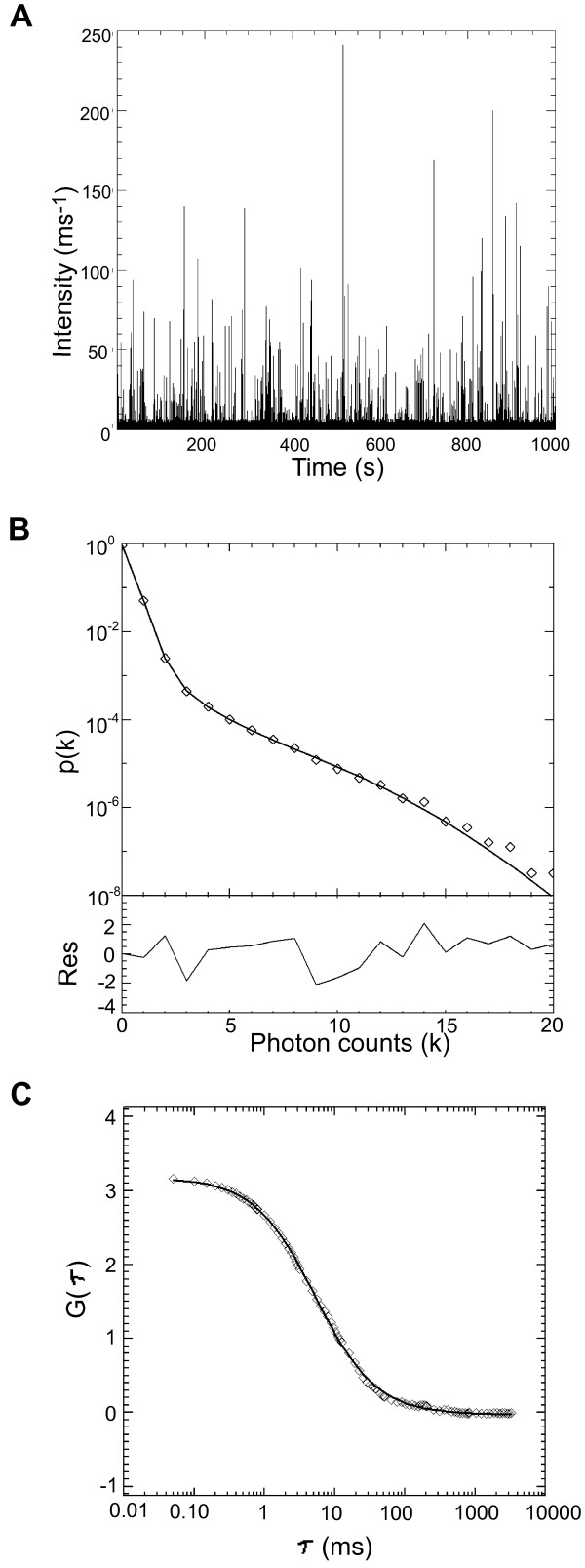
**Fluorescence fluctuation spectroscopy analysis of HTLV-1 Gag-based VLPs**. (A). The fluorescence intensity trace shows discrete peaks, which correspond to individual VLPs diffusing through the observation volume. (B). Experimental photon counting histogram (diamonds) of the VLP sample. A fit (solid line) of the histogram to a 2-species model with background identifies the concentration and Gag copy number of the VLPs. The presence of two species indicates the existence of heterogeneity in the Gag copy number of VLPs. A weighted average of the two species leads to an average Gag copy number of 530 per VLP. The first VLP species has a copy number of 270 and a concentration of 20.5 pM. The second VLP species, which is brighter than the first, has a copy number of 800 and a concentration of 6.5 pM. (C). A fit (solid line) of the autocorrelation function (diamonds) to a diffusion model identifies an average hydrodynamic diameter of 74 nm for the VLPs.

FFS provides information about the size of a particle through the autocorrelation function and the brightness and concentration of the particles through the photon counting histogram (PCH). Recent advances have expanded this technique to allow for the examination of protein oligomerization of larger complexes, including our recent analysis of HIV-1 particles [21]. In the current experiments, we performed measurements on the same cell culture supernatant from 293T cells transiently transfected with HTLV-1 Gag-EYFP and VSV-G expression constructs. The supernatant from these cells was clarified by a low-speed centrifugation to eliminate large cell debris, and then directly used for FFS analysis. Figure 5A shows a representative fluorescence intensity trace of a FFS experiment performed on the cell culture supernatant. The discrete fluorescence intensity spikes are produced by VLPs passing through the observation volume. This raw data was analyzed by fluorescence correlation spectroscopy to determine the average particle size from the autocorrelation function (Figure 5B). A fit to a single species diffusion model accurately describes the correlation function and identifies a diffusion time of 5.2 ms. This diffusion time corresponds to an average hydrodynamic diameter of 74 nm as determined by the Stokes Einstein relation. Repeating the measurement (*n *= 5) on independently prepared samples resulted in a mean diameter of 75 ± 4 nm for the VLPs.

The same raw data was analyzed with PCH analysis to determine the average copy number and concentration of VLP samples. A model assuming a single VLP brightness species leads to poor fits of the experimental PCH data (reduced χ^2 ^≥ 10). Including a second VLP brightness species into the fit model was required to reproduce the experimental data. A fit of the photon counting histogram to a 2-species model (reduced χ^2 ^= 1.5) is shown in Figure 5B. The presence of two brightness species indicates brightness heterogeneity in the VLP sample. In other words, the VLP particles passing through the laser excitation volume are not all of equal brightness, which gives rise to the additional brightness species. Each species *i *is characterized by its normalized brightness *b*_i _and average particle number *N*_i _in the observation volume. Note that the normalized brightness is the same as the Gag copy number of the VLP. It is illustrative to briefly ignore the brightness heterogeneity by calculating the average Gag copy number *b*_avg _of the VLP sample according to [22].

**Figure 6:**
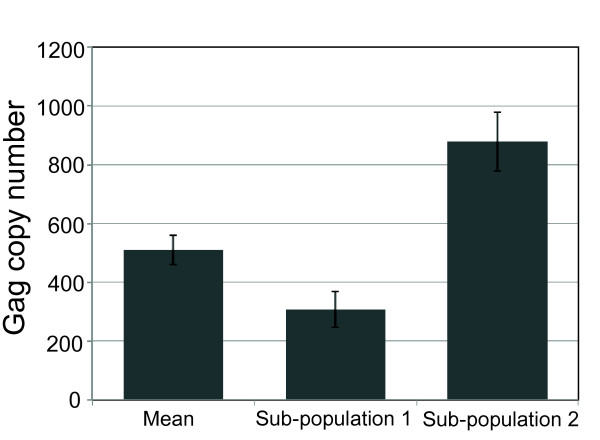
**Gag copy number of HTLV-1 Gag based VLPs**. The Gag copy number was determined by FFS analysis of several independent VLP samples (*n *= 5). The mean copy number per VLP is shown together with the corresponding copy number of the two subpopulations identified by FFS analysis. The error bars represent the standard deviation of the multiple measurements.

Based on measurements of several HTLV-1-like particle samples (*n *= 5) we determined an average Gag copy number per VLP of 510 ± 50 (Figure 6). To put this number into perspective, recall that a copy number of ~5000 Gag is required to completely fill the surface of a 140 nm HIV-1 VLP [5]. Thus, a maximum Gag copy number of ~1300 is expected for the smaller (~73 nm) HTLV-1 VLP assuming that both Gag proteins occupy a comparable surface area at the membrane. The observation of an average Gag copy number of 510 indicates that, on average, Gag at the membrane only covers about half of the available surface area.

The average Gag copy number was determined from the two brightness species identified by PCH analysis. Repeated measurements of multiple independent sample preparations confirmed the presence of the two species. Their brightness values, which typically varied very little across experiments, correspond to Gag copy numbers of *b*_1 _= 300 ± 60 and *b*_2 _= 880 ± 100 (Figure 6). The concentrations *N*_1 _and *N*_2 _varied from sample to sample, reflecting that total VLP production was dependent on sample-dependent factors, such as the initial cell density. However, the population fraction *f*_2 _= *N*_2_/(*N*_1_+*N*_2_) remained approximately constant for all measured samples, *f*_2 _= 19 ± 7%. Thus, a population of ~19% of the VLPs is associated with the higher Gag copy number. Note that a similar heterogeneity in Gag copy numbers has also been reported for HIV-1 VLPs [21].

## Discussion

Recent progress in cryo-TEM, cryo-ET and 3 D reconstruction has led to many major breakthroughs in our understanding of virus structure. For instance, the architecture of immature and mature HIV-1 [13,23,24], murine leukemia virus (MuLV) [25], and Rous sarcoma virus (RSV) [26] has been investigated in great detail. Although HTLV-1 was the first human retrovirus to be discovered [27,28], very little is known about HTLV-1 virion morphology. Progress in this area of HTLV-1 biology has been hampered due to the fragile nature of HTLV-1 proviral sequences as well as limited levels of viral gene expression and viral replication in tissue culture. HTLV-1 pathogenesis is typically observed decades after infection with low viral loads. In fact, studies have shown that HTLV-1 restricts its own gene expression via viral regulatory factors [29,30]. HTLV-1 has likely evolved such a replication strategy for immune escape. Furthermore, high AU-content of the retroviral genome may lead to instability during nuclear transport of mRNAs [31], which also contributes to the overall low level of viral gene and protein expression. In this study, we have designed a model system to study HTLV-1 Gag trafficking in cells and VLP production and morphology. The basis for this model system is a codon-optimized HTLV-1 Gag-EYFP construct, which can be readily amplified as a plasmid, expresses high levels of HTLV-1 Gag in mammalian cells, and robustly produces VLPs. This is the first model system developed for HTLV-1 for the study of virus particle assembly, release, as well as virus particle morphology.

While our model system does not express Gag in the context of a proviral sequence (i.e., codon-optimized and EYFP-tagged), our results indicate that the VLPs produced have the morphology of the authentic HTLV-1 immature particles. Furthermore, while the Gag trafficking pathways used by the HTLV-1 Gag in this model system may be different from that of Gag expressed from the provirus, the production of VLPs argues that the trafficking pathways are biological relevant since VLP production is the result of expression of the codon-optimized Gag-EYFP fusion. The altered Gag trafficking pathways could influence envelope incorporation into VLPs, though our cryo-TEM data revealed an abundance of VLPs with VSVG. The VLPs characterized in our study resemble immature HTLV-1 and can be readily observed in ultrathin sections of 293Ts transfected with pEYFP-N3 HTLV-1 Gag (Figure 1C). In addition, cell culture supernatants from 293Ts transiently transfected with pEYFP-N3 HTLV-1 Gag contain high levels of Gag-EYFP fusion proteins (Figure 1B), which provides second line of evidence for the production of VLPs. In the fraction of sucrose gradients containing the highly-fluorescent material, cryo-TEM reveals that these fractions are highly concentrated with VLPs (Figure 3A). Expression of EYFP alone in cells did not lead to the release of fluorescence in the cell culture supernatant (data not shown), arguing that we were specifically detecting the Gag-EYFP fusion in the VLPs.

We found that the intracellular localization of HTLV-1 Gag-EYFP was comparable to that of authentic Gag in HeLa cells (Figure 2). This implies, though does not formally prove, that there are similarities in the Gag trafficking pathway used by Gag-EYFP and authentic Gag. Among retroviruses, intracellular Gag polyproteins are thought to target and accumulate at membrane compartments prior to viral assembly. In the case of HIV-1, Gag is thought to primarily target specific domains of the plasma membrane where PI(4,5)P2 and cholesterol are enriched, though endosomal trafficking may also play a role. For HTLV-1, several studies have suggested the association of Gag with several markers found on the membranes of late endosomes and multivesicular bodies - these markers are also enriched at the plasma membrane [32-35].

Our cryo-TEM and FFS analysis determined that the average VLP diameter was 71 ± 20 nm and 75 ± 4 nm, respectively. As observed in other retroviruses, the size of HTLV-1-like particles varies greatly, ranging from 30 to 237 nm. According to the size distribution (Figure 3B), over 25% of the population is between 70-80 nm in diameter, indicating that HTLV-1 is smaller, on average, than previously believed. The average diameter of HTLV-1 has been shown to be anywhere from 95.1 ± 19.0 nm to 110.0 ± 15.5 nm depending on different types of staining used for TEM [19]. However, morphological details are lost with staining methods when the biological specimens are completely dehydrated. Examining frozen, hydrated samples via cryo-TEM reflects the native morphology of the viral particles. Moreover, FFS offers a unique way to determine the average hydrodynamic radius in the cell supernatant without any special treatment or preparation prior to FFS analysis. The use of two independent methods for determining VLP diameter provides a strong argument in favor of the relatively small particle diameter for the HTLV-1-like particles analyzed in our study.

We used FFS to also investigate Gag stoichiometry in the VLPs by performing brightness analysis of the FFS data. We determined that the average Gag copy number per VLP is ~510, which implies that only half of the available membrane surface is covered by Gag. Brightness analysis further revealed heterogeneity of the Gag copy number by identifying two brightness species. The presence of heterogeneity in the Gag copy number has also been observed for HIV-1 Gag-based VLPs [21]. Since FFS analysis involves an ensemble average over all measured VLPs, the information in the PCH curve only provides a rough approximation of the true Gag copy number distribution for the VLPs. Thus, the two brightness species identified by PCH analysis do not necessarily reflect two distinct populations of VLPs, but more likely reflect the analytical approximation of a broad distribution of Gag stoichiometries that approximately range from 300 to 880. PCH analysis also demonstrates that only ~20% of VLPs have high copy numbers.

Among the thousands of cryo-TEM images of VLPs examined in our study, we commonly observed particles that did not have electron density consistent with a Gag shell covering the entire membrane surface (Figure 3A). These results suggest that the majority of HTLV-1 particles analyzed contain an incomplete shell of Gag lattice. In the case of HIV-1, previous 3 D structural analyses revealed that most immature virions contain a continuous, but incomplete, hexameric arranged Gag shell, covering approximately 40-60% of the membrane surface [14,15,36]. The average copy number of Gag per particle was calculated to be approximately 2,400 ± 700 per immature particle. The Gag number increased significantly, however, when defects were introduced during budding [36]. In fact, the data is in agreement with our previous FFS study indicating that HIV-1 Gag stoichiometry ranges from 750 to 2,500 [21]. Since the mature core consists of only 1,000-1,500 molecules of CA [23], it is reasonable to believe that an equivalent number of Gag molecules are needed to form an immature particle. Our current study is the first to provide insights into the structural details for HTLV-1.

*In vitro *studies suggest that the HTLV-1 Gag shell is very likely to be organized differently compared to that of HIV-1 Gag [16-18]. When examining the cryo-TEM images of HTLV-1-like particles, we rarely observed a highly ordered Gag lattice next to the lipid bilayer (Figure 3A), a feature frequently observed in immature HIV-1 particles. The HTLV-1 particles analyzed in our study were fairly uniform in their overall inner density. Furthermore, in contrast to HIV-1, no defined peaks representing the CA or NC domains were found in the HTLV-1 radial density profile. The two peaks representing the lipid bilayers could be clearly determined (Figure 4), whereas the inner density profile appeared to be relatively flat. Since cryo-TEM images represent a two-dimensional projection of the virus particle, a more rigorous structural analysis, such as cryo-ET, is needed to further examine the protein organization in the HTLV-1-like particles.

In summary, we have developed the first efficient and robust model system for the analysis of HTLV-1 Gag cellular trafficking, virus particle assembly, release and particle morphology. This system will allow for significant advancements in understanding of the basic mechanisms of HTLV-1 replication - which has been severely hampered due to the limitations in studying HTLV-1 in tissue culture. Our study also represents the first description of immature HTLV-1 particles as well as quantitative measurements of particle size, Gag copy number, and an initial analysis of the HTLV-1 Gag lattice. Future application of cryo-electron tomography will aid in gaining greater insight into HTLV-1 particle morphology. A deeper understanding of the basic mechanisms involved in HTLV-1 particle assembly and morphology should help to enhance our global understanding of the basis of HTLV-1 particle infectivity, transmission and pathogenesis.

## Materials and methods

### Construction of codon-optimized HTLV-1 *gag-yfp *fusion

A codon-optimized HTLV-1 Gag gene was designed using the UpGene program [37] and synthesized by GenScript Co. (Piscataway, NJ). The synthetic HTLV-1 *gag *contains an optimal Kozak consensus sequence [38,39] at the 5' end of the gene: GCCACC**ATG**G (start codon in bold). Two restriction enzyme sites, Hind III and Bam HI, were also engineered into the 5' and 3' end of the gene, respectively, for sub-cloning purposes. For reporter gene construction, the artificial HTLV-1 *gag *was cloned into a pEYFP-N3 vector using the HindIII and BamHI restriction sites, creating pEYFP-N3 HTLV-1 Gag.

### Immunoblotting

293T cells were transiently transfected with the pEYFP-N3 HTLV-1 Gag construct using GenJet (SignaGen, Gaithersburg, MD) according to the manufacturer's instructions. Thirty-six hours post-transfection, cell pellets and supernatant were collected and lysates were prepared as previously described [40]. Lysates were subjected to electrophoresis on 12.5% polyacrylamide gels and transferred to nitrocellulose (Bio-Rad, Hercules, CA). HTLV-1 Gag polyprotein was detected with a primary mouse anti-HTLV-1 p24 antiserum (Abcam, Cambridge, MA) at 1:1500 dilution followed by a horseradish peroxidase-conjugated goat anti-mouse IgG (Thermo Fisher, Rockford, IL) at 1:5000 dilution. Gag polyprotein expression was detected with a ChemiDoc XRS system (Bio-Rad).

### Immunofluorescence and fluorescence microscopy

HeLa cells were grown on Lab-Tek II chamber slides (Fisher Scientific) and transfected with either the pEYFP-N3 HTLV-1 Gag construct or a HTLV-1 proviral clone (a kind gift from Dr. Marie-Christine Dokhelar) [41]. Thirty-six hours post-transfection, cells were washed twice with 1× PBS buffer and fixed with 4% paraformaldehyde for 20 min. For cells transfected with pEYFP-N3 HTLV-1, cells were washed three times after fixation, and stained for 5 min with 1 μg/ml DAPI (Sigma-Aldrich, St. Louis, MO) in 1× PBS containing 0.05% Triton X-100 (Sigma-Aldrich), then preserved using ProLong Gold antifade mounting regent (Invitrogen, Carlsbad, CA). For cells transfected with the HTLV-1 proviral clone, permeabilization was achieved by treating with 1× PBS containing 0.5% Triton X-100 for 2 min at room temperature following fixation. Cells were then washed three times and blocked with 1× PBS containing 5% normal donkey serum (Sigma-Aldrich) for 30 min. Primary mouse anti-HTLV-1 p24 antisera (Abcam) were diluted (1:150) in blocking solution and incubated with cells. After incubation for 2 hr at room temperature, cells were washed three times, followed by a second incubation for 1 hr at room temperature with diluted (1:250) Alexa Fluor 488-conjugated donkey anti-mouse IgG (Invitrogen). Prior to mounting, cells were washed five times and stained with DAPI as described above. Intracellular localization of Gag polyprotein was detected with an Olympus FV500 confocal laser scanning microscope. Optical sections of cells were collected with a Plan-Apo 60×/1.45 NA TIRFM objective at 1.5 zoom. The z-series were reconstructed using Olympus FluoView software.

### VLPs purification for cryo-TEM

293T cells were co-transfected with pEYFP-N3 HTLV-1 and a vesicular stomatitis virus G (VSV-*G*) protein (10:1) expression construct using GeneJet. Twenty-four hours post-transfection, the cell culture media was changed to a serum-free media and incubated for an additional 12 hr. In order to harvest VLPs, tissue culture supernatant was centrifuged at 3000 × g for 5 min to remove large cellular debris, then the supernatant was passed through an Amicon Ultra- 15 Centrifugal Filter Unit (100 KDa) (Millipore, Billerica, MA) to concentrate samples. The concentrated samples were then subjected to a 10-40% linear sucrose gradient prepared with a Gradient Master (BioComp, Fredericton, NB, Canada). Samples were then ultracentrifuged at 35,000 rpm for 30 min at 4°C using a SW55 Ti rotor. The VLP fraction was extracted and pelleted at 35,000 rpm, 4°C for 1.5 hr using a SW55 Ti rotor (Beckman). After centrifugation, the pellet was resuspended in 1× STE buffer (10 mM Tris-Cl, pH 7.4, 100 mM NaCl, 1 mM EDTA) at 4°C for 4 hr and then analyzed by cryo-TEM.

### TEM of transfected cells

293T cells were transfected with either pEYFP-N3 HTLV-1 or a HTLV-1 proviral clone as described above. Thirty-six hours post-transfection, cells were harvested and washed twice with 1× PBS followed by an additional wash in 0.1 M sodium cacodylate. To prepare thin sections, cell pellets were first fixed with 2.5% glutaraldehyde for 40 min and then washed three times with 0.1 M sodium cacodylate. After washing, the samples were post-fixed with 1% OsO_4 _for 30 min, followed by three rinses. The samples were then subjected to increasing concentrations of ethanol for dehydration. Immediately following the application of 70% ethanol, en bloc staining was added to the samples for 30 min before embedding in Epon 812 resin. Ultrathin sections (65 nm) were acquired and stained with uranyl acetate and lead citrate, then examined by electron microscopy using a JEOL 1200EX transmission electron microscope.

### Cryo-TEM of HTLV-1 VLPs and calculation of radial profile

A 3ul aliquot of the purified and concentrated HTLV-1 VLP sample preparation was applied to a glow-discharged c-flat holey carbon grid (Ted Pella, Redding, CA) and used for plunge freezing into liquid ethane [42] with a FEI Vitrobot MarkIII system. The frozen grids were then transferred to a FEI TF30 field emission gun transmission electron microscope at liquid nitrogen temperature. Images were recorded at a magnification of 40 k to 100 k at low-dose (~30 e/Å2) and 1 to 5 μm underfocus conditions using a Gatan 4 k by 4 k CCD camera.

In order to calculate the radial density profile, images of VLPs with spherical morphology were boxed using RobEM http://cryoem.ucsd.edu/programDocs/runRobem.txt. The center of each particle was determined using the program EMCORORG that calculates cross-correlation of each image with its 180° rotational image http://cryoem.ucsd.edu/programDocs. The radial profile for each particle was then calculated by computing the rotationally averaged density relative to the center of the particle. A group of 14 VLP images with a diameter in the range of 70-80 nm was used for calculation of the averaged radial profile. Each pixel in the image corresponds to a 3.0-Å spacing in the VLP. The defocus levels of these images were between 1.5-3.7 μm, which allows for visualization of both membrane leaflets of the viral membrane. The radial profile of each particle was first calculated to obtain the highest density position of the outer membrane leaflet. The radial profile of each particle was then linearly interpreted to match the position of the outer membrane to the averaged position (367-Å radius). The average radial profile and standard deviation were then calculated.

### VLP size measurements

Cryo-TEM images were analyzed using ImageJ software (NIH, Bethesda, MD). For each VLP, two perpendicular diameters were used to calculate the average diameter. The histogram was generated using GraphPad Prism 5 software (GraphPad, La Jolla, CA).

### VLP preparation and FFS experimental setup

293T cells were co-transfected with pEYFP-N3 HTLV-1 and a VSV-G expression construct (10:1) as described earlier. Aliquots of the cell culture supernatants used for subsequent cryo-TEM analysis were removed for parallel analysis by FFS. Thirty-six hours post-transfection, VLPs were harvested and clarified of cellular debris by low-speed centrifugation at 3000 × g for 5 min as well as passing through a 0.22 μm filter. The resulting clarified supernatants were then used for FFS measurements.

A mode-locked Ti:sapphire laser (Tsunami, Spectra-Physics, Mountain View, CA) pumped by an intracavity doubled Nd:YVO4 laser (Millenia, Spectra Physics) is the source of two-photon excitation. Experiments were performed on a modified Zeiss Axiovert 200 microscope (Thornwood, NY) as previously described [22]. Each FFS measurement collects data at a sampling frequency of 20 kHz for a duration of 20-30 min at an excitation wavelength of 905 nm. The viral particles are measured using a 63× C-Apochromat water immersion objective (N.A. = 1.2). The excitation power at the objective ranged from 0.1-0.4 mW. A volume of 200 μl of VLP solution was added to an 8-well Nunc Lab-Tek Chamber Slide mounted on the microscope. To avoid evaporation unused wells were filled with water and the slide was closed with a lid. Measurements were taken 10 μm above the bottom of the well.

### FFS data analysis

A brief description of the analysis method is provided here. A detailed discussion of FFS analysis of VLP samples can be found elsewhere [21]. The diffusion time was determined by fitting the calculated autocorrelation function to a single species diffusion model [43]. The ratio of diffusion time for the two samples is equated, according to the Stokes-Einstein relation, to the ratio of the hydrodynamic radii of the diffusing particles τ_D1_/τ_D2 _= r_1_/r_2_. The measured diffusion time of fluorescent spheres with a known radius of 50 nm serves as a reference to calculate the average diameter of the VLPs [44]. The FFS data was also fit to a 3-species PCH model with deadtime and afterpulsing corrections [45]. Each independent species in PCH is defined by its brightness *ε *and the average number *N *of particles in the optical observation volume. The particle number *N *is converted into a concentration after the observation volume is calibrated with a dye sample. One of the three species is required to take the auto-fluorescent background of the solution into account as discussed in a recent paper on HIV-1 VLPs [21]. This background species, which has a vanishingly small brightness, is included in every PCH fit and will not be reported. The other two species of the PCH model define the VLP sample. The presence of two brightness species indicates the existence of Gag copy number heterogeneity within the HTLV-1 VLP population as previously observed for HIV-1 VLPs [21]. PCH fitting was carried out by programs written in IDL 6.4 (Research Systems, Boulder CO). Error analysis of FFS data was carried out as previously described [46].

### FFS brightness calibration and experimental considerations

To avoid unwanted optical effects, all experiments are conducted in a power range where the fluorescence intensity of YFP scales quadratically with excitation power. We also confirmed that within this power range the average occupation number *N *remains constant, which establishes a constant optical observation volume. The brightness of a protein complex scales with the number of YFP-labels it contains [21]. The YFP copy number of a complex is determined by the normalized brightness *b *= *ε/ε*_YFP_, where *ε*_YFP _is the brightness of the YFP monomer and *ε *is the brightness of the complex. A calibration measurement of YFP brightness, *ε*_YFP_, is necessary to determine copy number. Because YFP brightness is difficult to determine at the low powers that the VLPs must be measured at to avoid saturation of the detector, the YFP brightness, *ε*_high_, is measured at a higher excitation power, *P*_high_. Conversion to the YFP brightness *ε*_low _for the low power *P*_low _of VLP experiments is achieved by using the relationship of power to brightness ε_low _= ε_high_(P_low_^2^/P_high_^2^). The data acquisition time for the VLP measurements was chosen such that at least 1000 VLPs passed through the observation volume, which is sufficient for statistical analysis of the data. All VLP measurements were performed at excitation powers that are free from saturation and bleaching artifacts [21]. The FFS experiments identified two brightness species for the VLP sample. The average normalized brightness *b*_avg _of the two species is determined by a non-linear relationship [22],

$$b_{avg}=\frac{b_{1}^{2}N_{1}+b_{2}^{2}N_{2}}{b_{1}N_{1}+b_{2}N_{2}},$$.

where *b*_i _and *N*_i _are the normalized brightness and the number of particles in the observation volume of each species.

## Competing interests

The authors declare that they have no competing interests.

## Authors' contributions

IFG, WZ, JLJ, KF, YC and JR carried out the experimental work, participated in the data analysis and interpretation, and contributed in the writing of the manuscript. WZ, JDM, YC and LMM conceived of the study, oversaw experimental design, data analysis, and interpretation as well as edited the manuscript. All authors read and approved the final manuscript.

## Supplementary Material

Additional file 1 **Supplemental Figure 1**. Low magnification cryo-TEM image of VLPs produced from 293T cells. Image provides another example of the types of particles observed by cryo TEM. Scale bar = 100 nm. Click here for file

## References

[B1] Proietti FA Carneiro-Proietti AB Catalan-Soares BC Murphy EL Global epidemiology of HTLV-I infection and associated diseases Oncogene 2005 24 6058 6068 10.1038/sj.onc.1208968 16155612

[B2] Gessain A Barin F Vernant JC Gout O Maurs L Calender A de The G Antibodies to human T-lymphotropic virus type-I in patients with tropical spastic paraparesis Lancet 1985 2 407 410 10.1016/S0140-6736(85)92734-5 2863442

[B3] Osame M Usuku K Izumo S Ijichi N Amitani H Igata A Matsumoto M Tara M HTLV-I associated myelopathy, a new clinical entity Lancet 1986 1 1031 1032 10.1016/S0140-6736(86)91298-5 2871307

[B4] Gheysen D Jacobs E de Foresta F Thiriart C Francotte M Thines D De Wilde M Assembly and release of HIV-1 precursor Pr55gag virus-like particles from recombinant baculovirus-infected insect cells Cell 1989 59 103 112 10.1016/0092-8674(89)90873-8 2676191

[B5] Briggs JA Simon MN Gross I Krausslich HG Fuller SD Vogt VM Johnson MC The stoichiometry of Gag protein in HIV-1 Nat Struct Mol Biol 2004 11 672 675 10.1038/nsmb785 15208690

[B6] Lanman J Lam TT Emmett MR Marshall AG Sakalian M Prevelige PE Jr Key interactions in HIV-1 maturation identified by hydrogen-deuterium exchange Nat Struct Mol Biol 2004 11 676 677 10.1038/nsmb790 15208693

[B7] Benjamin J Ganser-Pornillos BK Tivol WF Sundquist WI Jensen GJ Three-dimensional structure of HIV-1 virus-like particles by electron cryotomography J Mol Biol 2005 346 577 588 10.1016/j.jmb.2004.11.064 15670606 PMC6608732

[B8] Ono A Orenstein JM Freed EO Role of the Gag matrix domain in targeting human immunodeficiency virus type 1 assembly J Virol 2000 74 2855 2866 10.1128/JVI.74.6.2855-2866.2000 10684302 PMC111776

[B9] Ono A Ablan SD Lockett SJ Nagashima K Freed EO Phosphatidylinositol (4,5) bisphosphate regulates HIV-1 Gag targeting to the plasma membrane Proc Natl Acad Sci USA 2004 101 14889 14894 10.1073/pnas.0405596101 15465916 PMC522033

[B10] Bryant M Ratner L Myristoylation-dependent replication and assembly of human immunodeficiency virus 1 Proc Natl Acad Sci USA 1990 87 523 527 10.1073/pnas.87.2.523 2405382 PMC53297

[B11] Riffel N Harlos K Iourin O Rao Z Kingsman A Stuart D Fry E Atomic resolution structure of Moloney murine leukemia virus matrix protein and its relationship to other retroviral matrix proteins Structure 2002 10 1627 1636 10.1016/S0969-2126(02)00896-1 12467570

[B12] Usami Y Popov S Popova E Inoue M Weissenhorn W H GG The ESCRT pathway and HIV-1 budding Biochem Soc Trans 2009 37 181 184 10.1042/BST0370181 19143627

[B13] Fuller SD Wilk T Gowen BE Krausslich HG Vogt VM Cryo-electron microscopy reveals ordered domains in the immature HIV-1 particle Curr Biol 1997 7 729 738 10.1016/S0960-9822(06)00331-9 9368755

[B14] Briggs JA Riches JD Glass B Bartonova V Zanetti G Krausslich HG Structure and assembly of immature HIV Proc Natl Acad Sci USA 2009 106 11090 11095 10.1073/pnas.0903535106 19549863 PMC2700151

[B15] Wright ER Schooler JB Ding HJ Kieffer C Fillmore C Sundquist WI Jensen GJ Electron cryotomography of immature HIV-1 virions reveals the structure of the CA and SP1 Gag shells Embo J 2007 26 2218 2226 10.1038/sj.emboj.7601664 17396149 PMC1852790

[B16] Bertola F Manigand C Picard P Belghazi M Precigoux G Human T-lymphotrophic virus type I nucleocapsid protein NCp15: structural study and stability of the N-terminal zinc-finger Biochem J 2000 352 Pt 2 293 300 10.1042/0264-6021:3520293 11085921 PMC1221459

[B17] Christensen AM Massiah MA Turner BG Sundquist WI Summers MF Three-dimensional structure of the HTLV-II matrix protein and comparative analysis of matrix proteins from the different classes of pathogenic human retroviruses J Mol Biol 1996 264 1117 1131 10.1006/jmbi.1996.0700 9000634

[B18] Cornilescu CC Bouamr F Yao X Carter C Tjandra N Structural analysis of the N-terminal domain of the human T-cell leukemia virus capsid protein J Mol Biol 2001 306 783 797 10.1006/jmbi.2000.4395 11243788

[B19] Doultree JC Kiernan RE Lee JY Bowden DS McPhee DA Tokuyasu KT Marshall JA A new electron microscope positive staining method for viruses in suspension J Virol Methods 1992 37 321 335 10.1016/0166-0934(92)90032-9 1378851

[B20] Wills JW Craven RC Form, function, and use of retroviral gag proteins AIDS 1991 5 639 654 10.1097/00002030-199106000-00002 1883539

[B21] Chen Y Wu B Musier-Forsyth K Mansky LM Mueller JD Fluorescence fluctuation spectroscopy on viral-like particles reveals variable gag stoichiometry Biophys J 2009 96 1961 1969 10.1016/j.bpj.2008.10.067 19254556 PMC2717261

[B22] Chen Y Wei LN Muller JD Probing protein oligomerization in living cells with fluorescence fluctuation spectroscopy Proc Natl Acad Sci USA 2003 100 15492 15497 10.1073/pnas.2533045100 14673112 PMC307595

[B23] Briggs JA Wilk T Welker R Krausslich HG Fuller SD Structural organization of authentic, mature HIV-1 virions and cores Embo J 2003 22 1707 1715 10.1093/emboj/cdg143 12660176 PMC152888

[B24] Briggs JA Grunewald K Glass B Forster F Krausslich HG Fuller SD The mechanism of HIV-1 core assembly: insights from three-dimensional reconstructions of authentic virions Structure 2006 14 15 20 10.1016/j.str.2005.09.010 16407061

[B25] Nermut MV Mulloy B Consideration of the three-dimensional structure of core shells (capsids) in spherical retroviruses Micron 2007 38 462 470 10.1016/j.micron.2006.11.007 17223564

[B26] Briggs JA Johnson MC Simon MN Fuller SD Vogt VM Cryo-electron microscopy reveals conserved and divergent features of gag packing in immature particles of Rous sarcoma virus and human immunodeficiency virus J Mol Biol 2006 355 157 168 10.1016/j.jmb.2005.10.025 16289202

[B27] Poiesz BJ Ruscetti FW Gazdar AF Bunn PA Minna JD Gallo RC Detection and isolation of type C retrovirus particles from fresh and cultured lymphocytes of a patient with cutaneous T-cell lymphoma Proc Natl Acad Sci USA 1980 77 7415 7419 10.1073/pnas.77.12.7415 6261256 PMC350514

[B28] Poiesz BJ Ruscetti FW Reitz MS Kalyanaraman VS Gallo RC Isolation of a new type C retrovirus (HTLV) in primary uncultured cells of a patient with Sezary T-cell leukaemia Nature 1981 294 268 271 10.1038/294268a0 6272125

[B29] Gaudray G Gachon F Basbous J Biard-Piechaczyk M Devaux C Mesnard JM The complementary strand of the human T-cell leukemia virus type 1 RNA genome encodes a bZIP transcription factor that down-regulates viral transcription J Virol 2002 76 12813 12822 10.1128/JVI.76.24.12813-12822.2002 12438606 PMC136662

[B30] Clerc I Polakowski N Andre-Arpin C Cook P Barbeau B Mesnard JM Lemasson I An interaction between the human T cell leukemia virus type 1 basic leucine zipper factor (HBZ) and the KIX domain of p300/CBP contributes to the down-regulation of tax-dependent viral transcription by HBZ J Biol Chem 2008 283 23903 23913 10.1074/jbc.M803116200 18599479 PMC3259792

[B31] Schwartz S Felber BK Pavlakis GN Distinct RNA sequences in the gag region of human immunodeficiency virus type 1 decrease RNA stability and inhibit expression in the absence of Rev protein J Virol 1992 66 150 159 1727477 10.1128/jvi.66.1.150-159.1992 PMC238270

[B32] Blot V Perugi F Gay B Prevost MC Briant L Tangy F Abriel H Staub O Dokhelar MC Pique C Nedd4.1-mediated ubiquitination and subsequent recruitment of Tsg101 ensure HTLV-1 Gag trafficking towards the multivesicular body pathway prior to virus budding J Cell Sci 2004 117 2357 2367 10.1242/jcs.01095 15126635

[B33] Dorweiler IJ Ruone SJ Wang H Burry RW Mansky LM Role of the human T-cell leukemia virus type 1 PTAP motif in Gag targeting and particle release J Virol 2006 80 3634 3643 10.1128/JVI.80.7.3634-3643.2006 16537631 PMC1440400

[B34] Mazurov D Heidecker G Derse D HTLV-1 Gag protein associates with CD82 tetraspanin microdomains at the plasma membrane Virology 2006 346 194 204 10.1016/j.virol.2005.10.033 16325219

[B35] Mazurov D Heidecker G Derse D The inner loop of tetraspanins CD82 and CD81 mediates interactions with human T cell lymphotrophic virus type 1 Gag protein J Biol Chem 2007 282 3896 3903 10.1074/jbc.M607322200 17166843

[B36] Carlson LA Briggs JA Glass B Riches JD Simon MN Johnson MC Muller B Grunewald K Krausslich HG Three-dimensional analysis of budding sites and released virus suggests a revised model for HIV-1 morphogenesis Cell Host Microbe 2008 4 592 599 10.1016/j.chom.2008.10.013 19064259 PMC3454483

[B37] Gao W Rzewski A Sun H Robbins PD Gambotto A UpGene: Application of a web-based DNA codon optimization algorithm Biotechnol Prog 2004 20 443 448 10.1021/bp0300467 15058988

[B38] Kozak M Compilation and analysis of sequences upstream from the translational start site in eukaryotic mRNAs Nucleic Acids Res 1984 12 857 872 10.1093/nar/12.2.857 6694911 PMC318541

[B39] Kozak M An analysis of 5'-noncoding sequences from 699 vertebrate messenger RNAs Nucleic Acids Res 1987 15 8125 8148 10.1093/nar/15.20.8125 3313277 PMC306349

[B40] Wang H Norris KM Mansky LM Involvement of the matrix and nucleocapsid domains of the bovine leukemia virus Gag polyprotein precursor in viral RNA packaging J Virol 2003 77 9431 9438 10.1128/JVI.77.17.9431-9438.2003 12915558 PMC187409

[B41] Le Blanc I Rosenberg AR Dokhelar MC Multiple functions for the basic amino acids of the human T-cell leukemia virus type 1 matrix protein in viral transmission J Virol 1999 73 1860 1867 9971764 10.1128/jvi.73.3.1860-1867.1999 PMC104426

[B42] Baker TS Olson NH Fuller SD Adding the third dimension to virus life cycles: three-dimensional reconstruction of icosahedral viruses from cryo-electron micrographs Microbiol Mol Biol Rev 1999 63 862 922 table of contents 10585969 10.1128/mmbr.63.4.862-922.1999 PMC98980

[B43] Thompson NL Lieto AM Allen NW Recent advances in fluorescence correlation spectroscopy Current Opinion in Structural Biology 2002 12 634 641 10.1016/S0959-440X(02)00368-8 12464316

[B44] Wu B Chen Y Muller JD Fluorescence correlation spectroscopy of finite-sized particles Biophys J 2008 94 2800 2808 10.1529/biophysj.107.112789 18065475 PMC2267132

[B45] Hillesheim LN Müller JD The Photon Counting Histogram in Fluorescence Fluctuation Spectroscopy with Non-Ideal Photodetectors 2003 85 1948 1958 12944307 10.1016/S0006-3495(03)74622-0 PMC1303366

[B46] Müller JD Chen Y Gratton E Resolving Heterogeneity on the Single Molecular Level with the Photon-Counting Histogram 2000 78 474 486 10.1529/biophysj.107.112789 10620311 PMC1300655

