# Supplementary figures and images for: Biophysical analysis of HTLV-1 particles reveals novel insights into particle morphology and Gag stoichiometry
Source: Retrovirology. 2010 Sep 20;7:75. doi: 10.1186/1742-4690-7-75 (PMC2954917; doi:10.1186/1742-4690-7-75)

**Supplemental Figure 1**  
**Grigsby *et al***

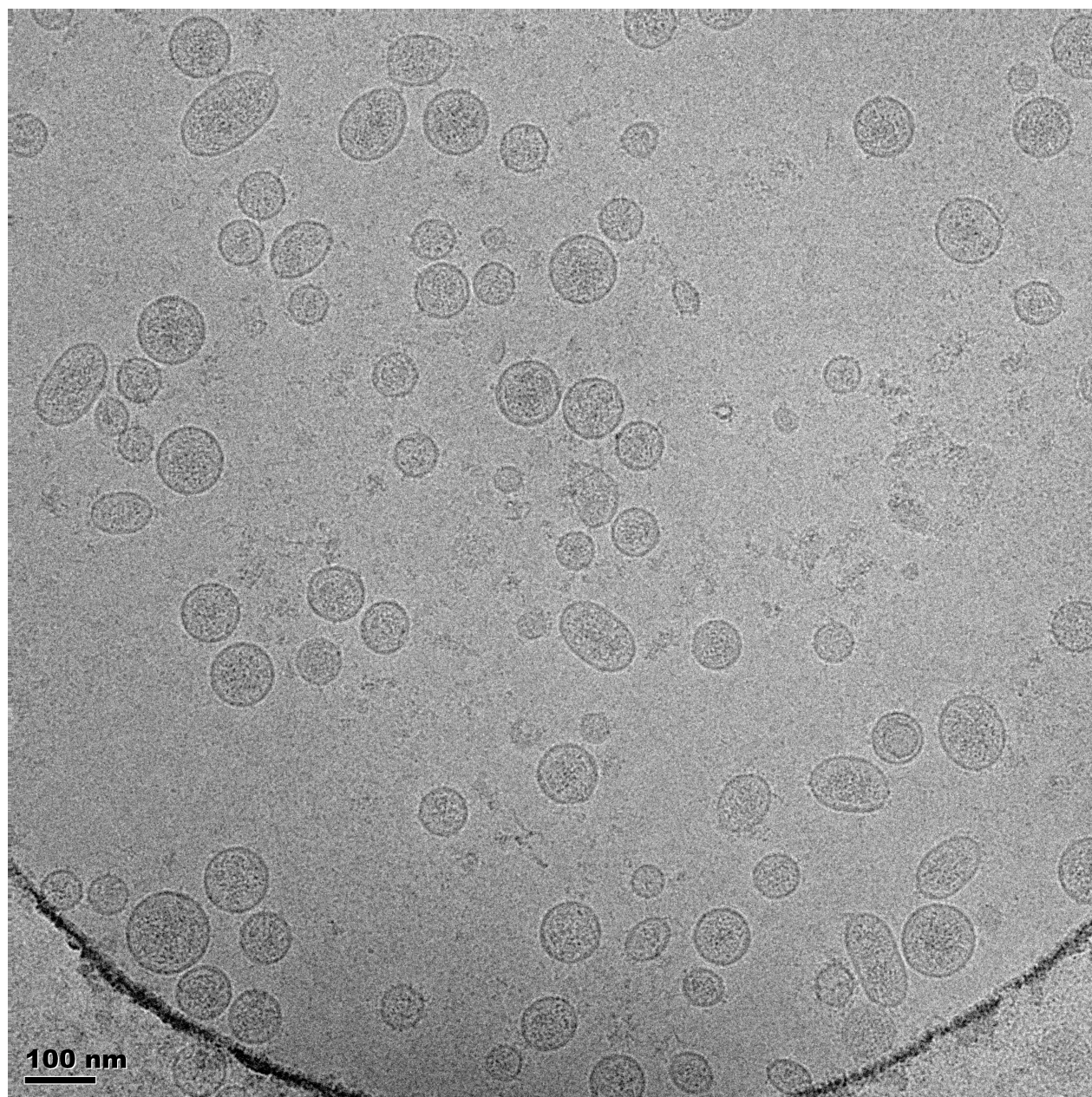

Supplement: Additional file 1 — Supplemental Figure 1. Low magnification cryo-TEM image of VLPs produced from 293T cells. Image provides another example of the types of particles observed by cryo TEM. Scale bar = 100 nm. [file 1742-4690-7-75-S1.PDF]
